# Euphorbia in Asthma and Bronchitis

**Published:** 1908-10-31

**Authors:** 


### EUPHORBIA IN ASTHMA AND BRONCHITIS.

Euphorbia is no new remedy, but it is one which has not been widely used, probably on account of the little attention that it receives in pharmacopœias. Whether there is any laboratory proof of the existence of an active principle in euphorbia preparations or not, those who have clinical experience of its use find that it is of distinct benefit in the treatment of the paroxysmal attacks of asthma, of bronchial catarrh, whooping cough, laryngitis with spasm of the glottis, paroxysmal dyspnœa, hay fever, and of angina pectoris.

Its mode of action is not well understood, though it is believed to exert its influence *via* the vagus nerve in some way. It is classed as an anti-spasmodic; some observers have even attributed an hypnotic action to it, but Dr. Artault, who has written an interesting article upon the subject in a recent issue of "Nouveaux Remèdes," believes that the apparently soporific effect of the drug is entirely an indirect one—sleep resulting in patients whose spasmodic dyspnœa or cough has been alleviated.

There are several varieties of euphorbia, of which Euphorbia pilulifera is, perhaps, the best known and most commonly employed. Dr. Artault states that the most active species is Euphorbia peplus, the entire plant being utilised.

The preparations that may be employed are the decoction, the tincture, and the extract. The decoction is made by boiling three-quarters of a teaspoonful of the dried entire plant, powdered and enclosed in a small muslin bag, in a pint of water, the volume being made up to one pint again at the end of the boiling. The dose of this decoction is an ordinary teacupful three times a day between meals. Larger doses than this are apt to cause vomiting and other signs of gastric disturbance; in some cases even the teacupful dose, when repeated, leads to faucial or pharyngeal irritation, in which case it is necessary to dilute the crude decoction until its acid taste is mitigated.

The tincture is given in 10 or 15 minim doses three times a day, or in smaller quantities at shorter intervals until the total that is being administered in 24 hours comes to about a drachm altogether. The extract similarly, up to a total of from 10 grains to half a drachm per diem.

